# Patient Satisfaction and Engagement in Therapeutic Activities in a Mental Health Rehabilitation Clinic

**DOI:** 10.1192/j.eurpsy.2026.11697

**Published:** 2026-07-31

**Authors:** F. S. Pricope

**Affiliations:** Rehabilitation centre, Tus Nua, Enniscorthy, Ireland

## Abstract

**Introduction:**

Therapeutic activities are essential in mental health rehabilitation, supporting recovery through social inclusion, physical wellbeing, creativity, and cognitive stimulation. While the UK’s QN Rehab recommends ~25 hours of structured activity weekly, no universal standard exists; locally, 3–5 hours per day (~15–25 hours/week) is used. Despite this, many patients attend infrequently, often due to lack of awareness, anxiety, or low confidence. This audit aimed to assess satisfaction and engagement, identify barriers, and incorporate patient feedback into programme development.

**Objectives:**

This aims to evaluate patient attendance and engagement in current activities, assess satisfaction with their variety, timing, and accessibility, and examine perceived benefits for mental health, social connection, and recovery. It also seeks to identify barriers to participation, such as anxiety, stigma, or lack of information, while gathering patient suggestions and preferences to inform the development of programmes that better meet their needs.

**Methods:**

**Design:** Cross-sectional audit (first cycle).

**Participants:** Inpatients with ≥2 weeks’ stay.

**Tools:**

- Patient Satisfaction Survey (attendance, preferences, perceived benefits, barriers).
- Staff Attendance Logs (objective participation data, engagement levels).
- Qualitative Feedback (free-text comments, optional interviews).
- **Analysis:** Combination of quantitative measures (attendance, satisfaction rates, benefit ratings) and qualitative themes (barriers, suggestions).
- **Duration:** Data collected over 3 months.

**Results:**

Many patients did not attend activities due to anxiety, low confidence, or lack of information, yet they suggested new options such as theatre games, karaoke, and opportunities to lead groups. Sporting activities, particularly team sports, were valued for enjoyment and social connection, while creative outlets like art and photography attracted strong interest, with some preparing for a community art festival. Yoga and meditation were consistently rated highly for relaxation and recovery, though some patients questioned the need for rehabilitation, underscoring the importance of addressing stigma and clearly communicating the benefits of participation.

**Conclusions:**

The first audit cycle identified gaps in patient engagement, often related to anxiety, stigma, and limited awareness of available activities. Patients expressed strong interest in expanding and diversifying programmes, particularly through creative, sporting, and patient-led initiatives.

Next steps involve introducing a short film to showcase the activity programme, developing patient-led groups to build confidence, expanding options such as sports, theatre, karaoke, and art, and integrating patients’ hobbies and interests into rehabilitation. A second audit cycle will be conducted in 3–6 months to assess whether these changes enhance satisfaction and engagement.

**Disclosure of Interest:**

None Declared

